# Reactive Astrocytes Derived From Human Induced Pluripotent Stem Cells Suppress Oligodendrocyte Precursor Cell Differentiation

**DOI:** 10.3389/fnmol.2022.874299

**Published:** 2022-05-06

**Authors:** Matthew D. Smith, Xitiz Chamling, Alexander J. Gill, Hector Martinez, Weifeng Li, Kathryn C. Fitzgerald, Elias S. Sotirchos, Dorota Moroziewicz, Lauren Bauer, Daniel Paull, Marjan Gharagozloo, Pavan Bhargava, Donald J. Zack, Valentina Fossati, Peter A. Calabresi

**Affiliations:** ^1^Department of Neurology, Johns Hopkins University School of Medicine, Baltimore, MD, United States; ^2^Department of Ophthalmology, Wilmer Eye Institute, Johns Hopkins University School of Medicine, Baltimore, MD, United States; ^3^The New York Stem Cell Foundation Research Institute, New York, NY, United States; ^4^Department of Genetic Medicine, Johns Hopkins University School of Medicine, Baltimore, MD, United States; ^5^Solomon Snyder Department of Neuroscience, Johns Hopkins University School of Medicine, Baltimore, MD, United States; ^6^Department of Molecular Biology and Genetics, Johns Hopkins University School of Medicine, Baltimore, MD, United States

**Keywords:** multiple sclerosis, astrocyte, oligodendrocyte, neurotoxicity, induced pluripotent stem cell (iPSC)

## Abstract

Astrocytes are instrumental in maintaining central nervous system (CNS) homeostasis and responding to injury. A major limitation of studying neurodegenerative diseases like multiple sclerosis (MS) is lack of human pathological specimens obtained during the acute stages, thereby relegating research to post-mortem specimens obtained years after the initiation of pathology. Rodent reactive astrocytes have been shown to be cytotoxic to neurons and oligodendrocytes but may differ from human cells, especially in diseases with genetic susceptibility. Herein, we purified human CD49f^+^ astrocytes from induced pluripotent stem cells derived from individual patient and control peripheral leukocytes. We compared TNF and IL1α stimulated human reactive astrocytes from seven persons with MS and six non-MS controls and show their transcriptomes are remarkably similar to those described in rodents. The functional effect of astrocyte conditioned media (ACM) was examined in a human oligodendrocyte precursor cell (OPC) line differentiation assay. ACM was not cytotoxic to the OPCs but robustly inhibited the myelin basic protein (MBP) reporter. No differences were seen between MS and control stimulated astrocytes at either the transcript level or in ACM mediated OPC suppression assays. We next used RNAseq to interrogate differentially expressed genes in the OPC lines that had suppressed differentiation from the human ACM. Remarkably, not only was OPC differentiation and myelin gene expression suppressed, but we observed induction of several immune pathways in OPCs exposed to the ACM. These data support the notion that reactive astrocytes can inhibit OPC differentiation thereby limiting their remyelination capacity, and that OPCs take on an immune profile in the context of inflammatory cues.

## Introduction

Astrocytes provide a range of homeostatic maintenance functions within the central nervous system (CNS) including neuron trophic support, synapse regulation, and blood–brain barrier integrity, among several others (Liddelow and Barres, 2017). In response to CNS pathology astrocytes can undergo reactive astrogliosis often diverging significantly from this homeostatic state (Schirmer et al., 2021). These reactive astrocytes undergo morphologic, transcriptomic, biochemical, metabolic, and functional changes that can support or limit CNS recovery (Faulkner et al., 2004; Brambilla et al., 2005; Fitch and Silver, 2008; Voskuhl et al., 2009; Brosnan and Raine, 2013; Linnerbauer and Rothhammer, 2020). Recent work in rodents has demonstrated that reactive astrocytes can adopt distinct activation states depending on the stimulus, including pro-inflammatory and pro-regenerative states defined by their transcriptomic profile and biologic functions (Zamanian et al., 2012; Liddelow et al., 2017). Pro-inflammatory reactive astrocytes defined in part by NF-κB activation and expression of complement-related factors (e.g., *C3* and *Serping1*) have a neurotoxic phenotype as a result of loss of the ability to promote neuronal survival and synaptogenesis, failed glutamate reuptake, secretion of neurotoxic lipid-mediators, altered chemoattractant functions, increased oxidative stress, and activation of T-cells and microglia (Liddelow et al., 2017; Barbar et al., 2020a; Giovannoni and Quintana, 2020; Guttenplan et al., 2021). Several studies have implicated reactive astrocytes in contributing to the pathogenesis of autoimmune demyelinating diseases including multiple sclerosis (MS) (Fontana et al., 1984; Wang et al., 2005; Ponath et al., 2018; Kamermans et al., 2019; Wheeler et al., 2020; das Neves et al., 2021). In addition to MS, pro-inflammatory reactive astrocytes are prominent in several other neurodegenerative disorders, including Alzheimer’s disease, Parkinson’s disease, and Huntington’s disease where they likely also contribute to neurodegeneration (Liddelow et al., 2017; Grubman et al., 2019; Al-Dalahmah et al., 2020; Absinta et al., 2021).

Current disease modifying therapies in MS prevent new inflammatory CNS lesions, but the majority of prior lesions remain chronically demyelinated and there are no clinically available therapies to promote remyelination after injury (Goldschmidt et al., 2009; Frischer et al., 2015). Oligodendrocyte precursor cells (OPCs) are critical for CNS remyelination as they are able to differentiate into new oligodendrocytes that can remyelinate axons (Dimou et al., 2008; Kang et al., 2010; Young et al., 2013; Hill et al., 2018). Astrocyte-oligodendrocyte interactions have been shown to be necessary in several models for promoting efficient remyelination (Franklin et al., 1991; Skripuletz et al., 2013; Monteiro de Castro et al., 2015; Houben et al., 2020; Lohrberg et al., 2020; Miyamoto et al., 2020), however, recent rodent data suggests pro-inflammatory reactive astrocytes can limit remyelination through inhibition of OPC migration, proliferation, and differentiation (Back et al., 2005; Wang et al., 2011; Liddelow et al., 2017; Lombardi et al., 2019; Miyamoto et al., 2020). This detrimental astrocyte-mediated inhibition of remyelination may contribute to failed remyelination and neuronal injury in both relapsing-remitting and progressive forms of MS as well as other inflammatory demyelinating diseases. Understanding the mechanisms of how astrocytes lose their homeostatic functions and become pathogenic effectors of chronic CNS neurodegeneration that contribute to failed remyelination is critical and may elucidate novel therapeutic strategies efficacious in promoting remyelination.

To that end, we aimed to study inflammatory astrocytes and their effect on OPC differentiation in a human cell model system. Access to human primary CNS cells has been largely limited by the availability of brain specimens, thus our knowledge of astrocyte and OPC/oligodendrocyte biology in myelination has mainly relied on rodent models. Human induced pluripotent stem cell (hiPSC) technology has recently been used to generate human astrocytes and other CNS cells *in vitro* (Li and Shi, 2020). We recently demonstrated that CD49f^+^ hiPSC-astrocytes display similar gene expression profiles to human primary astrocytes and perform critical astrocyte functions *in vitro* including glutamate uptake and support of neuronal growth and synaptogenesis (Barbar et al., 2020a). Similar to prior reports in rodents, these homeostatic functions are lost when hiPSC-astrocytes are polarized to a pro-inflammatory reactive phenotype (Barbar et al., 2020a).

Herein, we used hiPSC-astrocytes derived from people with MS and non-MS controls (NMSCs) to study the effect of pro-inflammatory cytokines on the astrocyte transcriptomes, and their secretomes on human embryonic stem cell (hESC) derived OPC differentiation and gene expression. This paradigm provides a novel human *in vitro* model to study astrocyte and OPC interactions and the potential to identify MS-specific glial phenotypes. Bulk transcriptome analysis of human hiPSC-astrocytes after cytokine stimulation (TNFα, IL1α) corroborates prior data in rodent astrocytes demonstrating a signature characterized by pro-inflammatory genes (Liddelow et al., 2017). We demonstrate that the pro-inflammatory human hiPSC-astrocyte secretome inhibits hESC-derived OPC differentiation and then we use bulk transcriptome analysis of human OPC-enriched cultures to identify significantly differentially regulated genes and pathways that may contribute to this failed remyelination phenotype.

## Results

### Astrocytes From Persons With Multiple Sclerosis and Non-Multiple Sclerosis Controls Respond Similarly to TNF + IL1α Stimulation

**FIGURE 1:**
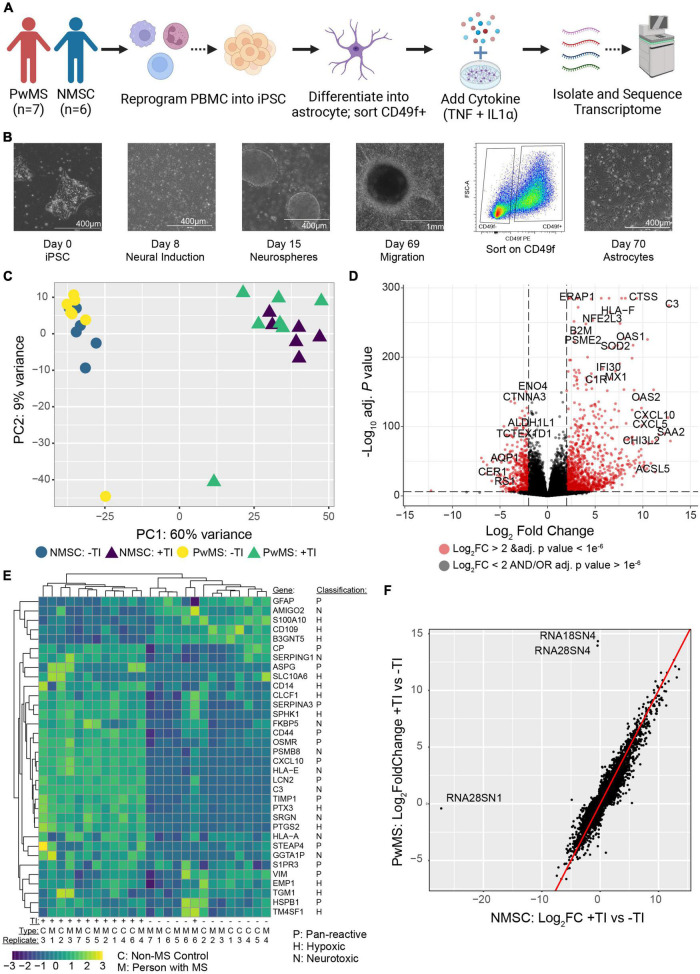
Human induced pluripotent stem cell derived astrocytes from PwMS and NMSC respond similarly to TNF + IL1α stimulation. **(A)** Schematic depicting experimental design. **(B)** Representative images showing stages of astrocyte differentiation from a PwMS. **(C)** Principle component analysis of top 500 most variable genes. **(D)** Volcano plot depicting results of differential expression testing comparing +TI treated astrocytes with –TI controls with a selection of the most significant genes labeled. **(E)** Heatmap showing clustering and relative expression of genes previously reported as characteristic of distinct sub-types of reactive astrocytes. Genes are classified as being previously reported as pan-reactive (P), associated with neurotoxic (N), or associated with hypoxic conditions (H). Samples are of Type C (NMSC) or M (PwMS). **(F)** *X*–*Y* scatterplot showing fold change of genes when comparing +TI to –TI conditions. Fold change of NMSC are on *X*-axis and of PwMS are on *Y*-axis. Red line has intercept of 0 and slope of 1. Most discrepant genes are labeled.

Given that C3+ neurotoxic astrocytes have been shown to be pathological in rodent models of MS (Gharagozloo et al., 2021), and enriched in persons with MS (PwMS) (Liddelow et al., 2017; Gharagozloo et al., 2021), we wished to determine whether induced pluripotent stem cell (iPSC) derived astrocytes from PwMS would respond differently to pro-inflammatory signals compared to NMSCs. Using hiPSCs reprogrammed from peripheral blood mononuclear cells (PBMCs) from seven PwMS and six NMSCs (seven controls were initiated but one failed quality control at the transcript stage) (Table 1), we differentiated them to a neural lineage using previously published methods (Paull et al., 2015; Barbar et al., 2020a; Figures 1A,B). After sorting on CD49f to enrich for astrocytes, we stimulated them with TNF and IL1α (TI) to promote a neurotoxic phenotype. Astrocytes were treated with or without TI for 48 h then RNA was collected and subjected to RNAseq analysis. Principle component analysis of the top 500 most variable genes showed the primary driver of variance was the addition of TI (Figure 1C). Differential expression testing between samples treated with TI and their paired untreated controls found 2228 upregulated genes and 1948 downregulated genes (Figure 1D and Supplementary Table 1). Examining relative expression of genes previously used to distinguish neurotoxic astrocytes from those associated with hypoxia in rodents showed an overlapping but distinct phenotype present in these human astrocytes, although the PwMS and NMSC samples clustered together suggesting no differences between them (Figure 1E). This lack of difference in TI response was further illustrated when we compared the fold change of genes in the NMSC group to the PwMS group relative to their −TI controls. The few genes that appear to respond differently are primarily ribosomal RNA present in an individual sample, likely due to incomplete ribosomal depletion in the RNAseq library preparation (Figure 1F and Supplementary Figure 1). Given women are more likely to develop MS than men, we assessed whether there was a difference in the response to +TI stimulation between astrocytes derived from females relative to those derived from males. Comparing the fold change of genes between the two sexes showed high similarity again except for a few extreme outliers driven by inefficient ribosomal RNA depletion in isolated samples (Supplementary Figure 2A). Seventy-five genes did differ moderately in their response to +TI stimulus in females compared to males (adjusted *p* < 0.05, Supplementary Figure 2B and Supplementary Table 2). While some of those were sex-linked genes (e.g., NLGN4Y), many were related to immune responses to cytokine signaling and were induced less in females relative to males (Supplementary Figures 2B–F).

**TABLE 1:** Demographics of hiPSC donors.

Group	Sample ID	Sex	Age at collection	Astrocyte RNAseq	OPC suppression	OPC nucleus count	OPC RNAseq
NMSC	051275	Male	43	Yes	Yes	Yes	No
NMSC	051282	Female	64	Yes	Yes	No	Yes
NMSC	051285	Male	64	Yes	Yes	Yes	No
NMSC	051290	Female	70	Yes	Yes	Yes	No
NMSC	051292	Male	74	Yes	Yes	No	Yes
NMSC	051313	Female	64	Yes	Yes	No	Yes
NMSC	051284	Female	60	No	No	No	Yes
PwMS	Bq0001	Female	65	Yes	Yes	Yes	No
PwMS	Bq0002	Female	65	Yes	Yes	Yes	No
PwMS	Bq0003	Male	75	Yes	Yes	Yes	No
PwMS	Bq0004	Female	51	Yes	Yes	No	Yes
PwMS	Bq0005	Male	60	Yes	Yes	No	Yes
PwMS	Bq0007	Female	45	Yes	Yes	No	Yes
PwMS	Bq0008	Female	51	Yes	Yes	No	Yes

*List of donors from whom hiPSC lines were derived and their demographic information. Last four columns indicate whether donor was used in said experiment.*

### Gene Ontology Term Enrichment Analysis Reveals Pathways Affected by TNF and IL1α Stimulation

**FIGURE 2:**
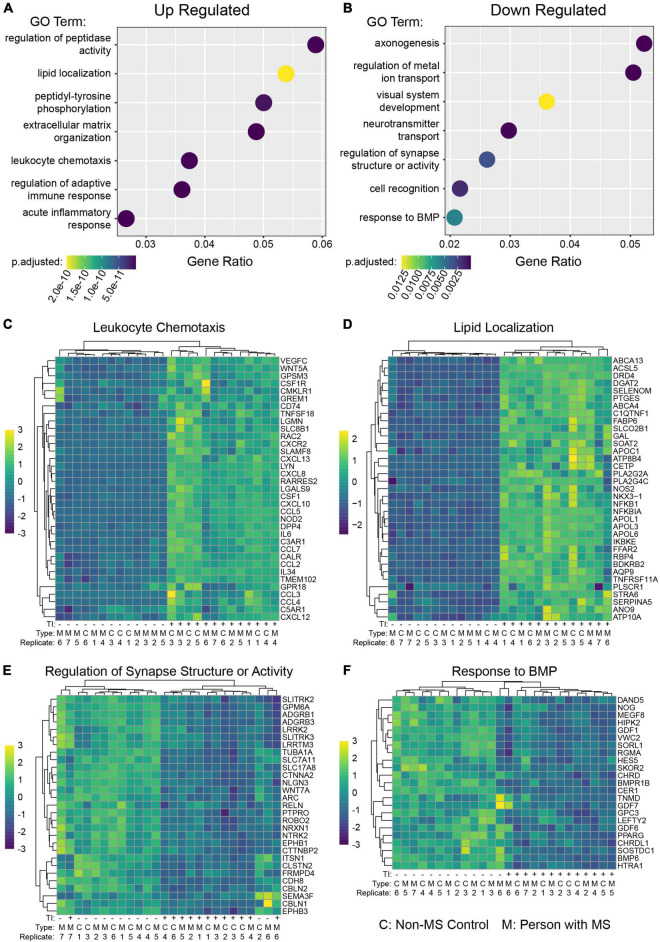
Gene ontology enrichment analysis of astrocytes following TI stimulation suggests shifts in function. GO enrichment analysis was performed on genes with a Log_2_ Fold Change value greater than 1 (up-regulated) or less than –1 (down-regulated) and adjusted *p*-value less than 0.05. A selection of enriched terms are shown in **(A)** (up-regulated pathways) and **(B)** (down regulated pathways), where the *X*-axis indicates the ratio of genes associated with that term to the total number of genes up or down regulated. BMP is bone morphogenic protein. Heatmaps showing relative expression values of genes associated with selected enriched GO terms in the up-regulated genes **(C,D)** and down-regulated genes **(E,F)**. Unsupervised clustering of genes is shown along left side and of samples along top.

To better understand the functional changes induced in human astrocytes following TI stimulation we performed gene ontology (GO) term enrichment analysis on genes found to be up-regulated or down-regulated when comparing the +TI to −TI conditions in all 13 cell lines. As expected, many of the enriched terms in the up-regulated genes were associated with inflammatory and anti-viral responses (Figure 2A and Supplementary Table 3), while many of the terms enriched in the down-regulated genes were associated with axonal support and nervous system development (Figure 2B and Supplementary Table 4). Further examination of individual genes associated with a select set of non-overlapping up-regulated (Figures 2C,D) and down-regulated (Figures 2E,F) terms again supported the prior results that PwMS and NMSC respond similarly to TI.

### Comparing TNF and IL1α Stimulated Human Induced Pluripotent Stem Cell Astrocytes to Previously Published Datasets

To compare the effect of TI on hiPSC astrocytes in this current dataset with previously published findings, we retrieved RNAseq data from rat neonatal astrocytes stimulated with TNF, IL1α, and C1q (TIC) for 24 h (Hasel et al., 2021) and hiPSC astrocytes stimulated with TIC for 24 h (Barbar et al., 2020a). As expected, there were many similarities between the three datasets, but also robust differences particularly between human and rat datasets that are not explained by difference in treatment (TI vs. TIC) or time (24 h vs 48 h) with only 42% (379/961) of up-regulated and 34% (468/1366) of down-regulated genes in rat also overlapping with either human dataset (Supplementary Figure 3). We also compared the transcriptomic profile of hiPSC astrocytes with or without TI stimulation to previously published single nucleus RNA sequencing (snRNA-seq) dataset derived from post-mortem human brain tissue including both controls and MS lesions (Absinta et al., 2021). Using non-parametric ranked correlations, we saw that all hiPSC astrocytes, regardless of TI stimulation, most resembled the inflamed and reactive astrocyte clusters (Supplementary Figure 4). The most pronounced effect of TI stimulation was to make them less like the non-reactive and senescent astrocyte clusters.

### TNF and IL1α Stimulated Astrocyte Conditioned Media Suppresses Differentiation of Human Oligodendrocyte Precursor Cells

**FIGURE 3:**
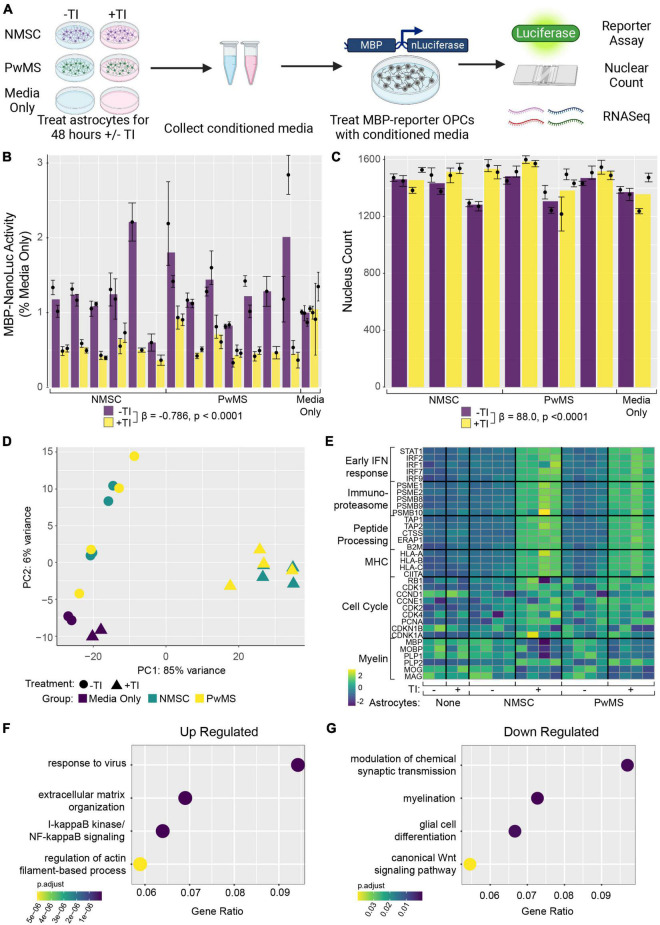
Conditioned media from TI stimulated astrocytes suppresses human OPC differentiation. **(A)** Schematic depicting experiment. **(B)** Barplot showing Nanoluciferase activity (normalized to media only control) on *Y*-axis. Each dot represents a well of hiPSC derived astrocytes whose media was placed onto five identical replicate wells of hOPC reporter cells. Columns represent mean of replicate astrocyte samples, dots represent mean of replicate OPC wells, and error bars represent standard error of the mean of replicate OPC wells. **(C)** Barplot showing nuclear counts following treatment with astrocyte CM, data presented in same format as in **(C)**. **(D)** Principle component analysis of hOPC reporter RNA libraries comparing samples treated with media only, media only with TI, and CM from NMSC and PwMS derived astrocytes treated with or without TI. **(E)** Heatmap showing relative expression of genes previously associated with inflammatory OPCs in hOPC reporter cells treated with astrocyte CM (and media only controls). Dotplots showing selected GO terms found to be enriched in up-regulated **(F)** and down-regulated **(G)** genes from hOPC reporter cultures treated with astrocyte CM stimulated with or without TI. Genes were identified as differentially expressed if they had adjusted *p*-value less than 0.05 and Log_2_ Fold Changes values greater than 0.5 or less than –0.5, respectively. Statistical analyses reported in **(B,C)** are result of linear mixed effect models.

In addition to their ability to kill neurons and mature oligodendrocytes, neurotoxic astrocytes have also been shown to suppress rodent OPC differentiation (Liddelow et al., 2017). In addition, hiPSC derived astrocytes with GFAP mutations have been shown to suppress human OPC differentiation in an *in vitro* model of Alexander’s disease (Li et al., 2018). To examine whether there was any difference between NMSC and PwMS in their oligo suppressive capabilities, we utilized a recently reported genetically modified reporter hESC line (Li et al., 2022). The reporter cells (from hereon called hOPC reporter cells) express rodent surface marker Thy1.2 and tdTomato under the control of the endogenous human PDGFRA promoter and a secreted Nanoluciferase (NLuc) under the control of the endogenous human myelin basic protein (MBP) promoter. This line allows for the enrichment of OPCs by selecting on Thy1 and then monitoring differentiation into oligodendrocytes by quantifying NLuc activity in the culture media (Li et al., 2022). We tested the ability of TI stimulated human astrocytes to suppress human OPC differentiation by collecting conditioned media (CM) from hiPSC derived astrocytes following 48 h of stimulation and treating hOPC reporter cells with that CM for 5–8 days (Figure 3A). To control for the possible effects of residual TI acting directly on hOPC reporter cells, media only controls with or without TI that were never in contact with astrocytes were used. The response to +TI CM did not differ between PwMS and NMSC (*p* = 0.48) or between females and males (*p* = 0.33). Across PwMS and NMSC, we found that CM from +TI treated astrocytes robustly suppressed hOPC reporter cell differentiation (Figure 3B, β_+*TI*_ = −0.786, standard error = 0.052, *p* < 0.0001). To determine whether the decreased amount of secreted luciferase was due to hOPC reporter cell death, we performed automated nuclear counts in a subset of the hOPC wells following treatment with astrocyte CM (Figure 3C). Performing similar analyses, we found no evidence of a different response between PwMS and NMSC (*p* = 0.47). We also saw no evidence that +TI CM reduced cell survival compared to −TI controls. In fact, we saw the opposite pattern, where hOPC wells treated with CM from +TI astrocytes had more nuclei (β_+*TI*_ = 88.0, standard error = 19.3, *p* < 0.0001), although the effect was modest.

### TNF and IL1α Stimulated Astrocyte Conditioned Media Causes Pronounced Transcriptomic Changes in hOPC Reporter Cells

Since the +TI CM was suppressing differentiation of human OPCs without killing them, we next performed RNAseq on the hOPC reporter cells following 72 h of treatment. Principle component analysis of the top 500 variable genes showed that hOPC reporter cells treated with CM from +TI treated astrocytes segregated separately from wells treated with CM from −TI treated astrocytes and the media only controls (Figure 3D). We then performed differential expression analysis to identify transcripts that are significantly altered in the +TI CM treated OPCs when compared to −TI CM as compared to the media only conditions. This analysis found 673 genes that were up-regulated and 205 genes that were down-regulated in +TI CM treated OPCs (Supplementary Table 5). Examining relative expression levels of a selection of genes we and others have previously reported to be differentially expressed in human and rodent OPCs following IFNγ stimulation (Chew et al., 2005; Kirby et al., 2019; Morales Pantoja et al., 2020), we saw consistency with upregulation of anti-viral response pathways, NF-κB signaling, and cell cycle progression and downregulation of myelin genes and glial differentiation (consistent with above luciferase reporter results). Indeed, GO term enrichment analysis in the up-regulated (Supplementary Table 6) and down-regulated (Supplementary Table 7) further supported these findings (Figures 3F,G).

## Discussion

Our results demonstrate that soluble factors released from pro-inflammatory reactive human astrocytes potently inhibit human OPC differentiation *in vitro*. The terminal differentiation of OPCs into oligodendrocytes coincides with exit of the cell cycle. Consistent with this, pro-inflammatory reactive astrocyte CM slightly increased OPC cell counts and induced expression of several pro-cell cycle genes (e.g., *CDK1*, *CDK2*, and *PDNA*). These findings support prior *in vitro* and *in vivo* rodent work showing that reactive astrocytes can impede remyelination through disruption of OPC migration, proliferation, and differentiation (Back et al., 2005; Wang et al., 2011; Liddelow et al., 2017; Lombardi et al., 2019; Miyamoto et al., 2020), and extend data from a recent study showing hiPSC reactive astrocyte CM is cytotoxic to human and rodent neurons (Barbar et al., 2020a). Bulk transcriptomic profiling of OPCs treated with pro-inflammatory astrocyte CM revealed increased inflammatory responses resembling interferon-gamma signaling seen in other contexts (Kirby et al., 2019), namely increased early interferon response, peptide processing, major histocompatibility complex (MHC), immunoproteasome, and cell cycle genes. These upregulated transcriptomic changes were associated with decreased expression of glial differentiation and extracellular matrix (ECM) regulation genes. Interestingly, this induction of antigen presentation pathways observed in OPCs is part of a transcriptomic profile we and others have described in inflammatory (i)OPCs *in vitro* and *in vivo* (Falcão et al., 2018; Kirby et al., 2019; Morales Pantoja et al., 2020). While MHC class I genes are most potently induced by interferon-gamma, other soluble factors likely participate in both inhibition of OPC differentiation and induction of iOPCs. Interestingly we do not see interferon-gamma induced by TI treatment (Supplementary Table 1) at the transcript level nor is it induced at the protein level in a companion paper in this issue (Fossati et al., 2022) thereby implicating other secreted signaling factors. Several candidate pro-inflammatory cytokines including IL6 and MIP-1a that were previously shown to be secreted by pro-inflammatory reactive astrocyte hiPSCs could mediate these observed effects (Barbar et al., 2020a). Several other potential OPC differentiation inhibitory factors have been identified including chondroitin sulfate proteoglycans, hyaluronans, fibrinogen, and fibronectin and many of these factors are produced by reactive astrocytes (Back et al., 2005; Stoffels et al., 2013; Keough et al., 2016; Petersen et al., 2017; Kirby et al., 2019).

Consistent with prior reports from primary rodent astrocytes and our own work in astrocyte hiPSCs (Liddelow et al., 2017; Barbar et al., 2020a), stimulation of astrocyte hiPSCs with TNF and IL1α induces a pro-inflammatory transcriptomic signature. Despite similar induction of a pro-inflammatory profile, there were significant differences in the transcriptomic signatures between these datasets, most strikingly between rodent and human astrocytes, highlighting the known limitations of extending or comparing rodent data to human model systems. In prior studies attempting to define reactive astrocyte subtypes, the nomenclature of “A1” vs. “A2” astrocytes based on transcriptomic profiles and functional readouts was used to refer to neurotoxic vs. neuroprotective or pro-inflammatory vs. anti-inflammatory reactive astrocyte states (Liddelow et al., 2017). While this dichotomous nomenclature akin to “M1” vs. “M2” in macrophage/microglia biology served as a useful framework in these early studies, reactive astrocytes do not consist of well-defined binary subtypes and likely can adopt many reactive states along several spectrums (Escartin et al., 2021). The reactive astrocyte hiPSCs in this study are transcriptionally characterized by upregulation of inflammatory, chemotactic, ECM, and lipid localization pathways and downregulation in bone morphogenic protein (BMP) response, synapse support and axonogenesis among other changes. Astrocytes with similar profiles have been identified in rodent models of MS and MS lesions suggesting a role for a similar pro-inflammatory subtype of astrocytes in MS pathology (Liddelow et al., 2017; Absinta et al., 2021; Clark et al., 2021). Comparing our hiPSC astrocytes to astrocyte transcriptomic clusters identified by snRNA-seq in post-mortem brain tissue from both controls and MS lesions (Absinta et al., 2021), we found that TI stimulation made hiPSC astrocytes less like the non-reactive and senescent clusters, suggesting a more reactive phenotype. Seemingly paradoxically, however, all hiPSC astrocytes, regardless of TI stimulation, most closely correlated with the inflamed and reactive astrocyte clusters. While this suggests some basal activation of hiPSC astrocytes *in vitro* and that TI treatment does not completely recapitulate the pro-inflammatory stimuli in MS brains *in vivo*, there are several potential confounders of these interpretations including comparing pooled snRNA-seq to bulk RNAseq, post-mortem tissue processing effects on gene expression, limitations of non-parametric analyses, and whether the abstract cluster titles precisely represent each astrocyte cluster. Further work is needed to identify and accurately define the spectrum of physiologic and pathologic astrocytes and how to recapitulate, functionally phenotype, and manipulate these spectrums *in vitro* and ultimately *in vivo* to identify novel astrocyte-targeting therapies.

While this study and prior work from other groups supports that some reactive astrocyte subtypes can impede remyelination (Back et al., 2005; Wang et al., 2011; Liddelow et al., 2017; Lombardi et al., 2019; Miyamoto et al., 2020), other studies have demonstrated that astrocytes are also necessary for OPC differentiation and efficient remyelination (Franklin et al., 1991; Skripuletz et al., 2013; Monteiro de Castro et al., 2015; Houben et al., 2020; Lohrberg et al., 2020; Miyamoto et al., 2020). This duality of reactive astrocytes either promoting or inhibiting remyelination is likely secondary to the specific reactive state of the astrocytes. In inflammatory demyelinating lesions such as in MS, the inflammatory milieu may drive reactive astrocytes within lesions toward subtypes that inhibit remyelination. These adverse reactive astrocytes may overwhelm any endogenous (or exogenous) pro-remyelination signals, including from potential OPC-targeted myelination therapies. This potential dominant inhibitory effect of pro-inflammatory reactive astrocytes may explain why several drugs that induce OPC differentiation and myelination of axons in pre-clinical studies including clemastine, bexarotene, and anti-LINGO-1 have had underwhelming results in MS clinical trials (Cadavid et al., 2017; Green et al., 2017; Brown et al., 2021). In support of this hypothesis, several OPC differentiation drugs have failed to rescue the inhibitory phenotype of chondroitin sulfate proteoglycans in an *in vitro* OPC remyelination model (Keough et al., 2016). However, modulation of the heparanome was shown to block interferon-γ-mediated inhibitory effects on OPC differentiation *in vitro* and recruitment *in vivo* (Saraswat et al., 2021), underscoring that inhibitory OPC signaling can be overcome in at least some paradigms. Effective remyelination strategies in MS may require concomitant antagonism of negative regulatory pathways and enhancement of positive regulatory pathways. Therapies that are able to reprogram reactive astrocytes toward pro-remyelination phenotypes may help promote remyelination and improve neuronal health in demyelinating lesions, particularly in conjunction with therapies that directly promote OPC differentiation and subsequent remyelination (Cadavid et al., 2017; Green et al., 2017; Brown et al., 2021).

While cytokine stimulation with TNF and IL1α reliably induced pro-inflammatory astrocyte hiPSCs with a secretome that inhibited OPC differentiation, we saw no difference in how hiPSC derived astrocytes from PwMS responded to the stimulation compared to NMSC. As would then be expected, we also observed no downstream differences between these groups in the transcriptomic profile or inhibition of differentiation of hiPSC OPCs exposed to pro-inflammatory astrocyte CM. There are several reasons this may be the case. Astrocyte hiPSCs used in this study were not from primary CNS cells and thus may not reflect inherent astrocyte epigenetic differences that depend on the CNS milieu. While automation of the derivation process has significantly improved the variance in hiPSC gene expression (Paull et al., 2015), hiPSCs require prolonged *in vitro* reprogramming that may mask inherent differences between cells derived from various subjects. Suprathreshold cytokine stimulation may also have overwhelmed or masked any potential inherent differences in response to inflammatory signaling. Additionally, if such inherent astrocyte differences are present this study may have been underpowered to detect such differences as the MS effect size may be small with considerable patient variability thus requiring an unfeasibly large hiPSC sample size.

There are several important limitations to the current study. MS is a highly heterogeneous disease and the small sample size limits generalizability of this result to all PwMS and importantly does not exclude the possibility of a subset unintentionally excluded from mediating a different response. Further, it will be important to examine the effects of MS gene variants expressed in astrocytes (e.g., rs7665090*^G^*) which could result in more robust astrocyte conditioned media (ACM) mediated pathology. In addition, it is possible that TI stimulation is not the critical pathway in MS astrocytes and that other cytokine induced pathways could yield different results. While the hOPC reporter cells were purified based on expression of PDGFRA, a marker specific to OPCs in this context, previous studies with this cell line have shown that some PDGFRA expressing cells can still trans-differentiate to astrocytes, so that the hOPC reporter cells likely contained some contaminating astrocytes. This limitation has little impact on the myelination and cell killing results, but is important to keep in mind when interpreting the RNAseq results, as we utilized a bulk strategy such that the transcriptomic changes would be influenced by contaminating astrocyte transcripts. In addition, while we had many biological replicates for the hiPSC astrocytes, the hOPC reporter line is derived from a single individual. So if there were differences present on the oligodendrocyte side of the astrocyte-oligodendrocyte interaction at the individual level, the study design implemented here would not detect them.

Despite the increased understanding of the central role of reactive astrocytes in several CNS neurodegenerative diseases, no current clinical therapies have been deliberately designed to target astrocyte-specific pathology. Manipulation of reactive astrocytes represents a largely novel therapeutic approach for treatment of both acute and chronic diseases of the CNS. Further work is critically needed to define the breadth of reactive astrocyte phenotypes, their role in promoting or preventing CNS pathology or repair, and the signaling pathways that ultimately can be targeted to reprogram neurotoxic, oligotoxic, and pro-inflammatory reactive astrocytes, among others, into more regenerative reactive subtypes.

## Materials and Methods

### Cells and Donors

Peripheral blood mononuclear cells were isolated from whole blood samples collected using gradient density separation using SepMate tubes (STEMCELL Technologies, 85450) and Lymphoprep (STEMCELL Technologies, 07801). PBMCs from PwMS were obtained from donors with the approval of the Johns Hopkins University Institutional Review Board. PBMCs from NMSC were obtained with approval by the New York Stem Cell Foundation’s external Institutional Review Board, WCG IRB (#20112091). All participants signed a consent and/or a repository consent as required by the overseeing IRB to allow their data and biospecimens to be repurposed.

### Human Induced Pluripotent Stem Cell Reprogramming

Peripheral blood mononuclear cells were thawed and recovered in StemPro-34 SFM Complete Medium (Thermo Fisher, 10639-011) supplemented with cytokines SCF (200 ng/μL, Thermo Fisher, PHC2111), Flt3 (200 ng/μL, Thermo fisher, PHC9411), IL3 (40 ng/μL, Thermo Fisher, PHC0034), and IL6 (40 ng/μL, Thermo Fisher, PHC0065), GlutaMAX (Thermo fisher, 35050-061) overnight as previously described (Zhou et al., 2015). The next day, a 96-well flat bottom plate was coated with Cultrex (HESC qualified Cultrex, Trevigen, 3434-0001-02) at 1:10 dilution and warmed for 1 h at 37°C. Cells were transferred into Cultrex coated 96-well flat bottom plates at 60K and 100K seeding density for reprogramming using CytoTune-iPS Sendai Reprogramming v2.0 Kit (Thermo Fisher, A16517) per the manufacturer’s recommendations, modified for cell number and plate format. After infection, cells were gradually transitioned to Freedom media (DMEM-F12 with Freedom-1 Supplement, Life Technologies, Custom) for 5 days post-infection with daily media changes on the NYSCF Global Stem Cell Array platform (Paull et al., 2015). Live cell surface staining using the iPSC marker Tra-1-60 (Tra-1-60 Antibody, Life Technologies) was performed 12–14 days post-transfection to identify reprogrammed cells. Successfully reprogrammed cell lines are consolidated in a 96-well Cultrex coated plate and stored in LN2 upon reaching confluency. Sendai reprogrammed iPS cell lines undergo enrichment and monoclonalization. This process utilizes FACS sorting on +CD56, +CD13, +Tra-1-60, +SSEA4 to first bulk sort newly reprogrammed lines to increase the iPSC population (enrichment) followed by single-cell sorting and machine learning augmented clonality assessment (Fischbacher et al., 2021) in order to select and consolidate monoclonalized iPSC lines. Monoclonalized iPSCs were then expanded *via* automation on the NYSCF Global Stem Cell Array platform for further quality control assays and then frozen into barcoded Matrix tubes in Synth-a-Freeze Cryopreservation Media at R500K cells/vial. All iPSC lines undergo rigorous quality control that includes a sterility check, mycoplasma testing, viability, karyotyping *via* Illumina Global Screening Array, SNP ID fingerprinting *via* Fluidigm SNPTrace, pluripotency and embryoid body scorecard assays *via* NanoString. iPSCs were maintained using Freedom media.

### Astrocyte Differentiation and Stimulation

Astrocytes were differentiated from iPSC precursors as previously described (Barbar et al., 2020a). Briefly, iPSCs were induced to neural stem cells (NSCs) within the first 12 days of differentiation. NSCs were expanded as floating neurospheres until day 30, when they were picked and plated onto poly-L-ornithine (0.1 mg/ml) and laminin (10 μg/ml)-coated dishes, at about 40 neurospheres per well of a 6-well plate. Cultures were maintained in PDGF medium for 4 weeks to allow for progenitor cells migration and differentiation toward astrocytes. PDGF medium composition: DMEM/F12, PenStrep (100×), 2-mercaptoethanol (1000×), MEM non-essential amino acids, N2 supplement (100×), B27 without VitA (50×), human insulin solution (25 μg/ml), PDGFaa (10 ng/ml), IGF-1 (10 ng/ml), HGF (5 g/ml), NT3 (10 ng/ml, T3 (60 ng/ml, biotin (100 ng/ml, cAMP (1 μM). Around day 70, cultures were dissociated through enzymatic digestion (Accutase, Thermo Fisher; A1110501) for 30 min at 37°C, and then filtered using a 70 μm filter (STEMCELL Technologies; 27260). Cells were spun at 300 g for 5 min at 4°C, resuspended in FACS buffer (PBS, 0.5% BSA, 2 mM EDTA, 20 mM Glucose) and incubated for 20 min with CD49f antibody (1:50; BD bioscience, 555736) for FACS purification. Sorted CD49f^+^ astrocytes were replated onto poly-L-ornithine/laminin-coated 24-well plates at 250K cells/well. Astrocytes were maintained in Glial medium (PDGF medium without the growth factors PDGF, IGF-1, HGF, NT3) for 24 h and then stimulated with TNFα (30 ng/ml; R&D system, 210-TA-020) and IL1α (3 ng/ml; Sigma, I3901) in Brainphys medium (STEMCELL Technologies, 05790) with B27 supplement minus antioxidants (Thermo Fisher, 10889038) for 48 h. CM from stimulated cultures and unstimulated controls were collected and frozen at −80°C until use, while cells were lysed for RNA isolation. For further details on astrocyte culture and stimulation to a neurotoxic reactive state see our previous publications (Barbar et al., 2020a,b).

### RNAseq

Cells (hiPSC derived astrocytes and reporter hOPCs) were lysed in RLT plus buffer and RNA was isolated using RNEasy Plus Micro kit (Qiagen 74034). Quality and quantity of RNA was determined using a NanoDrop and Fragment Analyzer. Of the 14 pairs of samples from iPSC derived astrocytes, 13 were sufficient to proceed to library preparation. Of the 10 pairs of samples from hOPC reporter cells, all 10 were sufficient to proceed. RNAseq libraries were prepared using Illumina Stranded Total RNA Prep with Ribo-Zero Plus kit (Illumina 20040525). Libraries were sequenced using 100 bp paired end configuration on Illumina NovaSeq. Quality of sequencing data was checked with FastQC (Andrews, 2017) then MultiQC (Ewels et al., 2016) and no data was discarded or trimmed. Transcript level estimated counts were acquired with Salmon (v1.4.0) (Patro et al., 2017) using default options but with the gcBias flag enabled. For selective alignment, the entire genome was used as a decoy sequence (Srivastava et al., 2020). RefSeq GRCh38.p12 was used as the reference genome and transcriptome (O’Leary et al., 2016). Estimated transcript level counts were then imported into R (v4.1.2), adjusted for transcript length, and aggregated to gene level using tximeta (v1.12.3) (Love et al., 2020). Count normalization and differential expression testing was done with DESeq2 (v1.34.0) (Love et al., 2014). For the astrocyte experiment, the contrast was (+TI to −TI) with blocking for individual in the model. For the OPC experiment, the contrast was [(astrocytes +TI to astrocytes −TI)–(media_only +TI to media_only −TI)] again with blocking for individual in the model. Log fold change was adaptively shrunk using the ashr package (v2.2-47) in DESeq2 (Stephens, 2017). Genes were considered differentially expressed if they had an adjusted *p*-value less than 0.05 and a Log_2_ Fold Change greater than 1 or less than −1 in the astrocyte experiment and greater than 0.5 or less than −0.5 in the hOPC reporter experiment. Volcano plot in Figure 1 was generated using the EnhancedVolcano package (v1.12.0) (Blighe et al., 2021). Gene Ontology enrichment analysis was conducted with clusterProfiler (v4.2.1) (Yu et al., 2012) using the enrichGO function with default options against the “Biological Process” GO library (Ashburner et al., 2000; Gene Ontology, 2021). Heatmaps were generated with pheatmap (v1.0.12) using the rlog scaled data derived from DESeq2. Unsupervised clustering in the heatmaps was performed using complete linkage method.

### Comparisons With Previously Published Transcriptomic Data

RNAseq data for rat astrocytes treated with TIC was obtained from the Gene Expression Omnibus (GEO) under accession GSE165069 (Hasel et al., 2021). All conditions were retrieved and included in the model but differential expression testing was only done to compare TIC vs. vehicle. RNAseq data from previously published hiPSC derived astrocytes treated with TIC was obtained from the Synapse open source platform under accession syn21861229 (Barbar et al., 2020a). Analysis of both previous datasets followed same method as above. Rat data was quantified using mRatBN7.2 assembly (Howe et al., 2021). For comparing between datasets, genes with 1:1 orthologs from human to rat were identified with biomaRt (Durinck et al., 2009) and only those genes were used in the cross-species comparisons. For pathway enrichment of genes differentially expressed exclusively in rat or human, genes were only included if they were differentially expressed in both human datasets (for those exclusive to human) or in neither (for those exclusive to rat). To avoid including genes that were differentially expressed but that did not meet the fold change threshold, a gene was only considered exclusive if the ortholog in the other species either (1) did not have an adjusted *p*-value less than 0.05 or (2) the Log2 Fold Change was in the wrong direction, i.e., >0 if being compared to down regulated genes in other species or <0 if being compared to up regulated genes in other species. The snRNA-seq data was obtained from GEO under accession GSE180759 (Absinta et al., 2021). Using Seurat (v4.1.0) (Satija et al., 2015), cells previously identified as astrocytes in the original publication were subsetted and the top 1000 highly variable genes were identified using log-normalized data. Pseudobulk gene expression levels for each cluster were calculated based on the previously published cluster identities for each cell. Spearman correlation coefficients were calculated for each hiPSC astrocyte sample and snRNA-seq cluster pairing using all 20,622 genes detected in both datasets or only the top 1000 highly variable genes in snRNA-seq astrocyte dataset with R (v4.1.2).

### hOPC Reporter Cell Line Differentiation

Previously published human ESC reporter cell lines for OPC (Chamling et al., 2021) and oligodendrocyte (Li et al., 2022) differentiation were used for the study. The reporter cell line used for this study contains PDGFRa-P2A-tdTomato-P2A-Thy1.2 and MBP-P2A-secNLuc reporters. In these reporter cells, upon *PDGFRa* expression, tdTomato as well as Thy1.2 protein product are produced. Since Thy1.2 is a surface protein, it migrates to the cell surface, allowing the PDGFRa expressing cells to be immunopurified *via* Thy1.2 antibody conjugated magnetic microbeads (Chamling et al., 2021; Li et al., 2022). In addition, since the endogenous *MBP* promoter drives the expression of NLuc (Promega) that is secreted into the cell culture media, MBP expression can be quantitated by measuring NLuc activity in the cell culture media (Li et al., 2022).

The hESC reporters were differentiated into OPCs by following the previously published detailed protocol (Douvaras and Fossati, 2015). Briefly, hESCs were plated on Matrigel and maintained in mTeSR plus. Neural differentiation and spinal cord patterning was induced through dual SMAD inhibition (SB431542, 10 μM and LDN193189, 250 nM) and 100 nM all-trans retinoic acid for 8 days. From day 8 to day 12, differentiating cells were maintained in neural induction media supplemented with RA (100 nM) and SAG (1 mM). At day 12, cells were lifted and cultured in low-attachment plates to support sphere aggregation. At day 30, spheres were plated into poly-L-ornithine/laminin-coated dishes in a media supplemented with B27 (Thermo Fisher, 12587010), N2 supplement (Thermo Fisher, 17502048), PDGF-AA (221-AA-10, R&D systems), neurotrophin-3, HGF (294-HG-025 R&D systems), and T3. Following this differentiation protocol, PDGFRa-tdTomato/Thy1.2+ cells are visible as early as day 45 of differentiation, PLP1-GFP and NanoLuc activity is detected from day 60 onward.

### hOPC Treatment With Astrocyte Condition Media and hOPC Reporter Assay

The hOPC Nluc reporter assay was performed as previously described (Li et al., 2022). Day 85 differentiating culture was MACS purified with Thy1.2 (CD90.2 microbeads, Miltenyi Biotech) to enrich for PDGFRA-tdTomato+ OPCs (Chamling et al., 2021). 1.5K cells/well of the OPCs were plated in PLO-laminin coated 384 well plates in 50 μL of glial differentiation media. Two days after plating the cells, the culture media was replaced with glial media containing 1/3 of the astrocyte condition media (CM) (i.e., 30 μL of astrocyte CM plus 60 μL of glial media, total 90 μL per well of a 384 well plate) using integra ViaFlow 384.

Myelin basic protein-Nluc activity was measured using NanoGlo luciferase assay reagents (Promega N1150). Briefly, 20 μL of cell culture media from an hOPC culture was collected in a 384 well plate. NanoGlo reaction mix was prepared by mixing Nano-Glo^®^ Luciferase Assay Substrate and Nano-Glo^®^ Luciferase Assay Buffer (1:50), and the reaction mix was further diluted (1:1) with water. To measure Nluc activity, 5 μL of the diluted reaction mix was added to each well containing the 20 μL culture media. Nluc activity was measured as relative light unit (RLU) with a microplate reader (ClariOstar, BMG LABTECH) using the preloaded settings for Nano luciferase and 1 s exposure.

### hOPC Nuclear Counts

Cells were fixed with 4% PFA, washed with PBS and either stored in 4°C or immediately imaged. Prior to imaging, Hoechst 33342 nucleic acid stain (Thermo Fisher) was diluted to 1:10,000 in PBS and added to each well containing the cells. Image was captured with High-Content Imager (Cellomics CX7) using 10× magnification and nine fields to cover the entire well. A built-in algorithm of the ArrayScan image analysis software, which is a part of the Cellomics CX7 (Thermo Fisher Scientific), was used to count the total number of nuclei per well.

### Statistical Analysis

Statistical analyses were performed with R version 4.1.2. Normalized NLuc and nuclear count results (Figure 3) were analyzed with linear mixed-effects models. Normalized NLuc readings or nuclear counts were included as the response, condition (+TI or −TI) and group (NMSC or PwMS) as fixed effects, and technical replicates nested within individuals as random effects. To test whether there was a difference in the effect of +TI between the NMSD and PwMS groups, models were also fitted included an interaction term between condition and group, but no significant difference was observed (likelihood ratio test *p* > 0.05).

## Data Availability Statement

The datasets presented in this study can be found in the Gene Expression Omnibus (https://www.ncbi.nlm.nih.gov/geo/) under accession GSE196575.

## Ethics Statement

The studies involving human participants were reviewed and approved by Johns Hopkins University Institutional Review Board and New York Stem Cell Foundation’s External Institutional Review Board. The patients/participants provided their written informed consent to participate in this study.

## Author Contributions

MS, XC, MG, PB, DZ, VF, and PC conceived and designed the experiments. MS, XC, HM, WL, DM, LB, and DP performed the experiments and collected the data. MS, XC, AG, WL, KF, and ES analyzed the data. MS, AG, XC, and PC wrote the manuscript. All authors reviewed and suggested improvements to manuscript.

## Conflict of Interest

ES reports: scientific advisory board and/or consulting for Viela Bio, Horizon Therapeutics, Genentech, and Alexion; speaking fees from Alexion, Viela Bio, and Biogen. PC reports: PI on grants to JHU from Genentech and Principia; consulting fees from Biogen, Disarm Therapeutics (now owned by Lilly), and Avidia Technologies (now Vaccitech). The remaining authors declare that the research was conducted in the absence of any commercial or financial relationships that could be construed as a potential conflict of interest.

## Publisher’s Note

All claims expressed in this article are solely those of the authors and do not necessarily represent those of their affiliated organizations, or those of the publisher, the editors and the reviewers. Any product that may be evaluated in this article, or claim that may be made by its manufacturer, is not guaranteed or endorsed by the publisher.
