# Supplementary material for: Reactive Astrocytes Derived From Human Induced Pluripotent Stem Cells Suppress Oligodendrocyte Precursor Cell Differentiation
Source: Front Mol Neurosci. 2022 May 6;15:874299. doi: 10.3389/fnmol.2022.874299 (PMC9120968; doi:10.3389/fnmol.2022.874299)
Supplement: Supplementary file 1 [file Data_Sheet_1.docx]

**Supplementary Material**

**
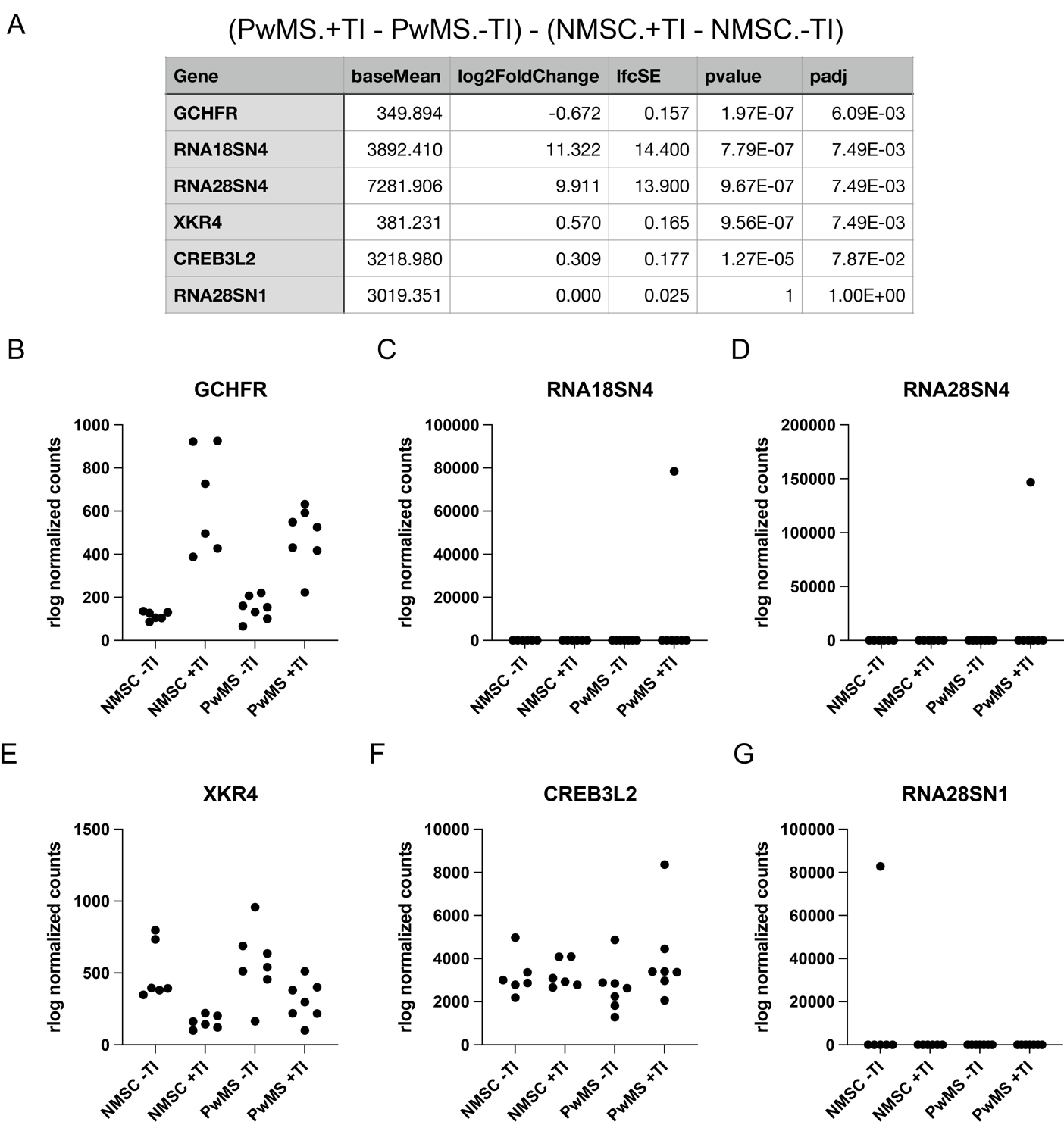
**

**Figure S1. Genes responding to +TI differently in PwMS compared to NMSC. (A)** Table showing results of differential expression testing comparing stimulation with TI in astrocytes from PwMS compared to stimulation in NMSC. baseMean is mean count across all samples. **(B-G)** Dot plots showing rlog normalized counts for genes listed in **(A)** separated by group (NMSC or PwMS) and condition (-TI or +TI)

**
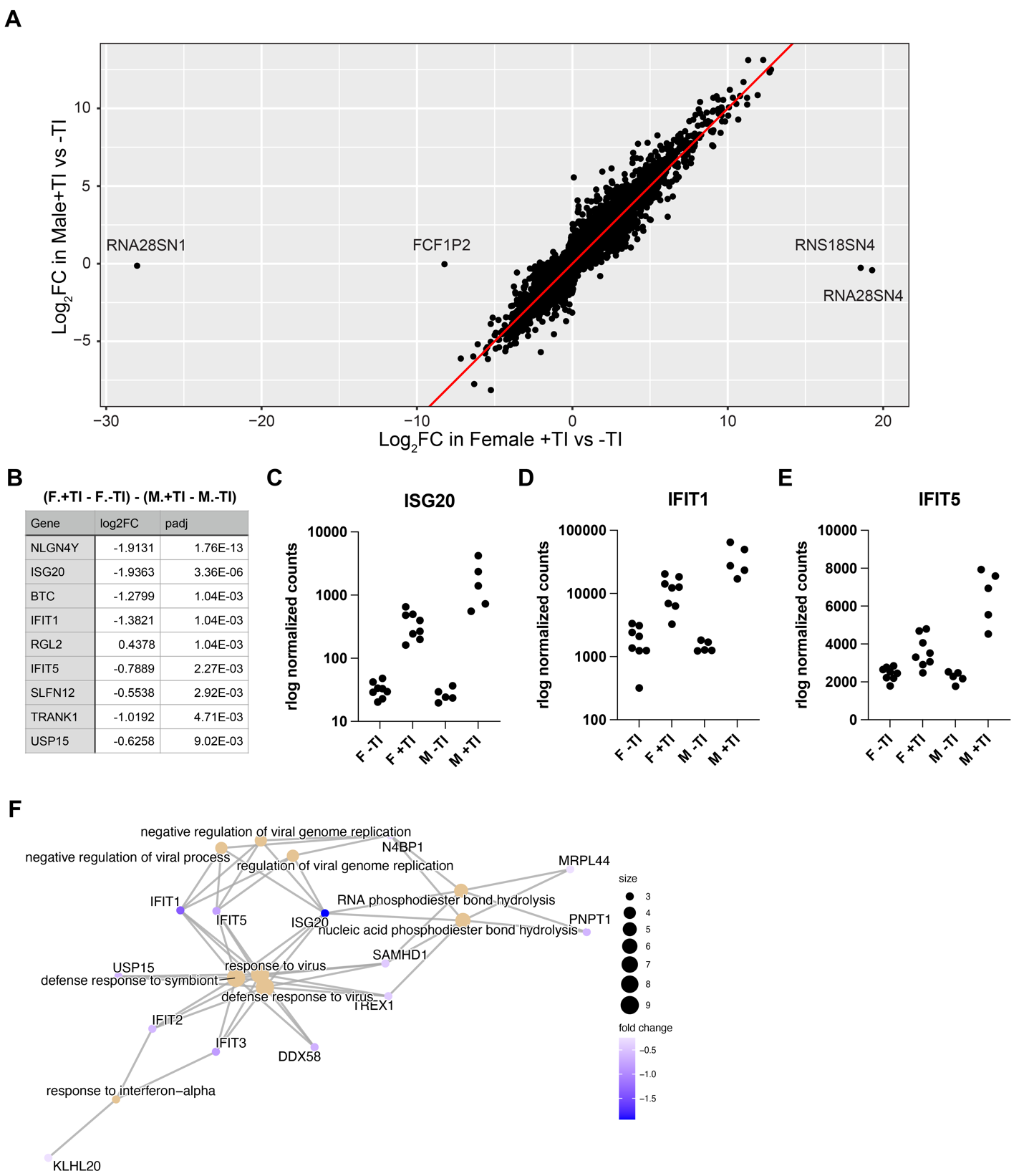
**

**Figure S2: Genes responding to +TI differently in hiPSC astrocytes derived from female and male donors. (A)** X-Y scatterplot showing fold change of genes when comparing +TI to -TI in female (x-axis) and male (y-axis) astrocytes. Red line has intercept of 0 and slope of 1. Most discrepant genes are labeled. **(B)** Table showing selection of top genes differentially expressed in astrocytes from female (F) donors in response to +TI relative to astrocytes from male (M) donors. **(C-E)** Dot plots showing rlog normalized counts for some of the genes listed in **(B)**. **(F)** Network plot showing selection of GO terms enriched in the genes differentially expressed in astrocytes from female donors relative to male donors in response to +TI where GO terms are brown nodes and genes that contribute to them are linked with edges. Size of terms indicate number of genes associated with term in DEG list, color of gene nodes indicates log_2_fold change (astrocytes from female donors relative to male donors -- negative numbers indicates decrease in gene expression in astrocytes derived from female donors).

**
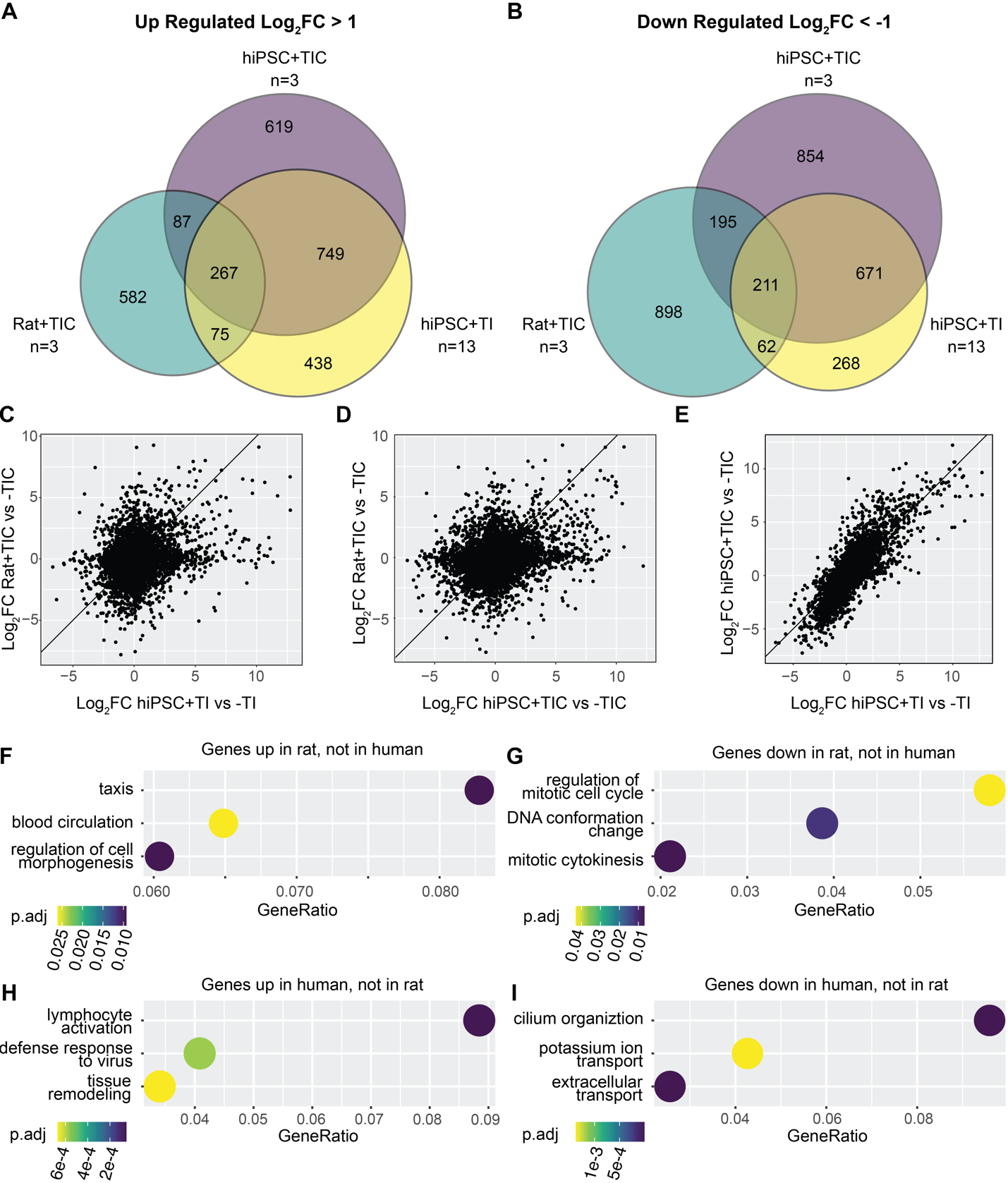
**

**Figure S3. Similarities and differences in response to cytokine stimulation in human and rat astrocytes. (A, B).**Venn diagrams showing overlapping and non-overlapping genes upregulated (Log2FC > 1 and p.adj < 0.05, **A**) and downregulated (Log2FC < -1 and p.adj < 0.05, **B**) in 3 transcriptomic datasets: hiPSC astrocytes stimulated with TNF+IL1a (TI) for 48 hours (current study), hiPSC astrocytes stimulated with TNF+IL1a+C1q (TIC) for 24 hours (Barbar et al), and rat astrocytes stimulated with TIC for 24 hours (Hasel et al). Only genes with 1:1 orthologues are included. **(C-E)**X-Y scatterplots showing Log2FC of genes in one dataset on x-axis and another dataset on y-axis with absolute line of x=y. **(C)**shows hiPSC+TI vs rat+TIC. **(D)** shows hiPSC+TIC vs rat+TIC. **(E)** shows hiPSC+TI vs hiPSC+TIC. **(F-I).** Dotplots showing selected results of GO term enrichment analysis of genes up in rat dataset and not in either human dataset **(F)**, down in rat dataset and not in either human dataset **(G)**, up in both human datasets and not in rat **(H),**or down in both human datasets but not in rat **(I).**Gene ratio is number of genes associated with term divided by total number of differentially expressed genes. For **(F-I)**, genes were considered exclusive to species if in other species they either had p.adj > 0.05 or Log2FC in opposite direction (ie >0 when compared to down-regulated genes or <0 when compared to up-regulated genes).


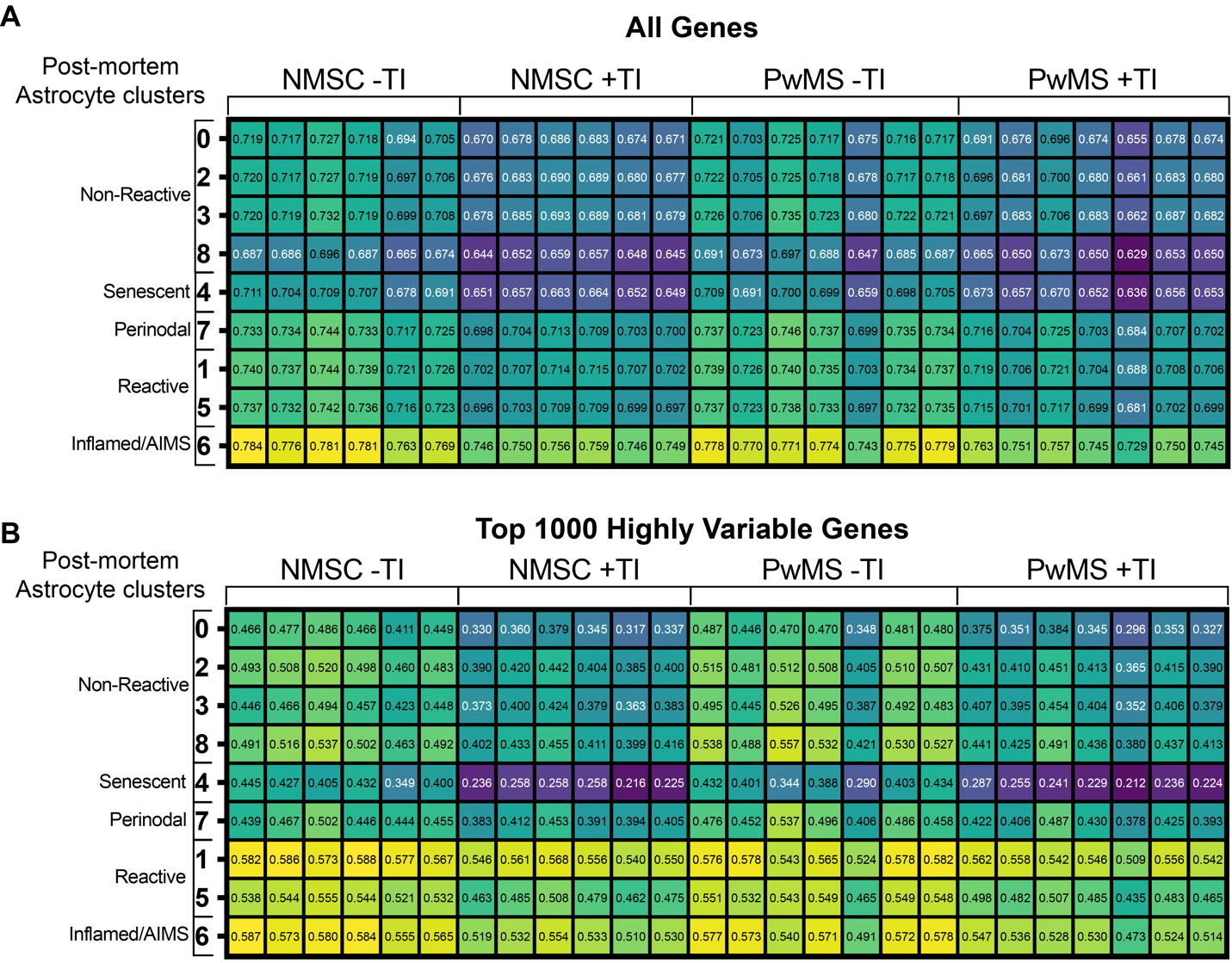


**Figure S4. Comparison of hiPSC derived astrocyte transcriptomes with post-mortem snRNA-seq astrocyte clusters.** Previously published snRNA-seq on astrocytes from controls and MS lesions (Absinta et. al) was obtained and pseudobulk averages for each identified astrocyte cluster were calculated. Transcriptomic profiles of hiPSC astrocytes with and without TI stimulation were compared with each cluster by calculating Spearman correlation coefficients which are shown in each cell of the tables. **(A)** Correlation using all 20,622 genes detected in both datasets. **(B)** Correlation using only the top 1000 highly variable genes in snRNA-seq dataset. Rows represent identified clusters with associated numbers and classifications as presented in published snRNA-seq dataset . Columns represent hiPSC sample:treatment combinations.

**Table S1: Differential Expression testing results comparing +TI astrocytes to –TI astrocytes.**

**Table S2: Differential Expression testing results comparing (+TI F astrocytes vs -TI F astrocytes) – (+TI M astrocytes vs -TI M astrocytes).**

**Table S3: GO Term enrichment analysis of genes upregulated in +TI vs –TI astrocytes.**

**Table S4: GO Term enrichment analysis of genes downregulated in +TI vs –TI astrocytes.**

**Table S5: Differential Expression testing results comparing hOPC reporter cells treated with +TI astrocyte conditioned media compared to –TI astrocyte conditioned media vs. +TI media only compared with –TI media only.**

**Table S6: GO Term enrichment analysis of genes upregulated in Table S4.**

**Table S7: GO Term enrichment analysis of genes downregulated in Table S4.**
